# Superresolution concentration measurement realized by sub-shot-noise absorption spectroscopy

**DOI:** 10.1038/s41467-022-28617-w

**Published:** 2022-02-17

**Authors:** Korenobu Matsuzaki, Tahei Tahara

**Affiliations:** 1 Molecular Spectroscopy Laboratory, RIKEN, 2-1 Hirosawa, Wako, 351-0198 Japan; 2 Ultrafast Spectroscopy Research Team, RIKEN Center for Advanced Photonics (RAP), RIKEN, 2-1 Hirosawa, Wako, 351-0198 Japan

**Keywords:** Quantum metrology, Optical spectroscopy

## Abstract

Absorption spectroscopy is one of the most widely used spectroscopic methods. The signal-to-noise ratio in conventional absorption spectroscopy is ultimately limited by the shot noise, which arises from the statistical property of the light used for the measurement. Here we show that the noise in absorption spectra can be suppressed below the shot-noise limit when entangled photon pairs are used for the light source. By combining broadband entangled photon pairs and multichannel detection, we realize the acquisition of sub-shot-noise absorption spectra in the entire visible wavelength. Furthermore, we demonstrate the strength of sub-shot-noise absorption spectroscopy for the identification and quantification of chemical species, which are two primary aims of absorption spectroscopy. For highly diluted binary mixture solutions, sub-shot-noise absorption spectroscopy enables us to determine the concentration of each chemical species with precision beyond the limit of conventional absorption spectroscopy. That is, sub-shot-noise absorption spectroscopy achieves superresolution in concentration measurements.

## Abstract (web summary)

Here, the authors use entangled photon pairs as the light source for absorption spectroscopy and demonstrate sub-shot-noise spectra in the entire visible wavelength region. They quantify chemical species in highly diluted solutions with precision beyond the limit of conventional spectroscopy.

## Introduction

Absorption spectroscopy is one of the simplest and most widely used spectroscopic methods. In this method, one irradiates light onto a sample of interest and measures the intensity of the transmitted light. Despite the simplicity of the experiment, it can yield a large variety of information about the sample, from the vibrational modes and the electronic structure of the constituent molecules^1^ to the atomic species comprising the sample and their local environment^2^, depending on the wavelength range of the light used in the measurement (i.e., infrared, visible and ultraviolet, or X-ray).

In practice, an absorption spectrum is obtained by performing two kinds of measurements, namely sample and reference measurements. In the sample measurement, photons are shone onto a sample, and the transmitted photons are detected in a wavelength-sensitive manner. In the reference measurement, which is usually done in parallel to the sample measurement, the same number of photons are sent directly to another detector without a sample. By writing the number of photons detected in the sample measurement as ${N}^{S}$ and that in the reference measurement as ${N}^{R}$, the absorbance $A$ of the sample is given by

1 $$A=-{{{\log }}}_{10}\frac{{N}^{S}}{{N}^{R}}.$$

An absorption spectrum of the sample is obtained by plotting the absorbance $A$ as a function of wavelength.

In this experimental procedure, it is assumed that the number of incident photons in the sample measurement can be chosen to be exactly the same as in the reference measurement. This assumption, however, cannot be strictly satisfied in actual measurements because of the statistical property of the classical light such as a laser^3,4^: No matter how carefully the number of incident photons is adjusted in the two measurements, the best is to balance the statistical average of the photon number per unit time, and the photon number at each instant inevitably deviates from one another because it fluctuates randomly following the Poisson statistics. This mismatch of the incident photon number in the reference and sample measurements results in the noise in the measured absorption spectra, which is called the shot noise. Defining the noise as the standard deviation of absorbance, the noise $\delta A$ can be expressed in terms of the absorbance $A$ as

2 $$\delta A=\sqrt{{{{{{\rm{Var}}}}}}\left(A\right)}=\sqrt{{{\langle }}{\left(A-\left\langle A\right\rangle \right)}^{2}{{\rangle }}}.$$

Here, ${{{{{\rm{Var}}}}}}\left(A\right)$ is the variance of $A$, and $\langle \cdot \rangle$ denotes the statistical average. Following this definition, the shot noise ${{{{{\rm{\delta }}}}}}{A}_{{SN}}$ in absorption spectra is given by (see Supplementary Note 4 for the derivation)

3 $${{{{{\rm{\delta }}}}}}{A}_{{SN}}=\frac{1}{{{{{{\rm{ln}}}}}}10}\sqrt{\frac{1}{{N}^{S}}+\frac{1}{{N}^{R}}}.$$

The shot noise is usually considered to be unavoidable because it arises from the intrinsic property of the light itself. That is, the lower bound for the noise level in an absorption measurement is determined by the shot noise ${{{{{\rm{\delta }}}}}}{A}_{{SN}}$ (commonly known as the shot-noise limit).

Contrary to this common belief, there is actually a chance to overcome this shot-noise limit when an exotic state of light, i.e., nonclassical light, is used as the light source. Nonclassical light, or quantum light, is a state of light that cannot be described within the framework of classical electrodynamics. Examples of nonclassical light include single photons, squeezed light, and entangled photon pairs^3,4^. The use of such nonclassical light^5^ for spectroscopic purposes is a rather unexplored topic. Nevertheless, considering that the advances of optical spectroscopy so far have been largely supported by the advent of new light sources, lasers in particular^6^, the use of nonclassical light may lead to a breakthrough in the further development of spectroscopic techniques.

Several types of nonclassical light sources that achieve a noise level below the shot-noise limit, i.e., sub-shot-noise operation, are reported. The Sandoghdar group, for instance, generated intensity-squeezed light using a single molecule with a near-unity spontaneous emission quantum efficiency^7^. Here, the idea is that the single molecule emits exactly one photon each time it is excited by a train of excitation pulses, resulting in an extremely regular stream of emitted photons. The Walmsley group and Bowen group have also obtained intensity-squeezed light based on degenerate optical parametric amplification, and they applied it to the measurements of stimulated emission^8^ and stimulated Raman signals^9^, respectively. Its two-beam variant, i.e., two-mode squeezed light, where the intensity difference between the two beams is squeezed below the shot-noise limit, has been realized as well, for example, by the Leuchs group using nondegenerate optical parametric amplification in a nonlinear crystal^10^ and by the Agarwal group using a four-wave mixing process in an atomic vapor^11^. In the latter study, the feasibility of absorption measurements at the sub-shot-noise level was also demonstrated.

Using entangled photon pairs, on the other hand, the Rarity group has done pioneering works on sub-shot-noise absorption measurements, in which the noise in the measurement is suppressed below the shot-noise limit^12,13^. More recently, the Genovese group has demonstrated sub-shot-noise absorption imaging^14,15^. Sub-shot-noise absorption spectroscopy has also been reported by the Matthews group^16^. However, their spectral window was only 10 nm in width in the near-infrared region (from 807 nm to 818 nm), and the measurement involved time-consuming wavelength sweeping. Thus, compared with conventional absorption spectroscopy, there was a substantial limitation in the measurement procedure as well as obtainable spectra.

In this study, we report the development of broadband sub-shot-noise absorption spectroscopy with multichannel detection. This method enables the acquisition of absorption spectra in the entire visible wavelength (from 450 nm to 650 nm) in a single measurement without wavelength sweeping. In addition, at each wavelength in the obtained absorption spectra, the noise is suppressed well below the shot-noise limit. Thus, our broadband multichannel sub-shot-noise absorption spectroscopy yields absorption spectra in a wide wavelength range comparable to the one obtained with conventional absorption spectroscopy, while the noise level is suppressed below the fundamental limit of the conventional method. Using the developed method, we further show how the noise suppression realized in this study enhances the precision of the identification and quantification of the chemical species in a sample, which are two primary aims of performing absorption spectroscopy measurements.

## Results

### Principle of operation

As the light source for the sub-shot-noise absorption measurements in this study, entangled photon pairs in the visible wavelength are used, which can be generated via the spontaneous parametric down-conversion (SPDC) process^3,4^. In this SPDC process, ultraviolet pump light is introduced into a nonlinear crystal such as β-barium borate (BBO), and a high-energy ultraviolet photon is split into two paired photons with lower energy. The entangled photon pairs generated in this manner have three key properties that are utilized to realize broadband multichannel sub-shot-noise absorption spectroscopy.

First, as is clear from the name, all the photons exist as pairs, meaning that every photon is accompanied by a partner photon. Thus, if we split each photon pair into two, we can have two groups of photons containing exactly the same number of photons without any statistical uncertainty. It is thus possible to beat the shot-noise limit in absorption spectroscopy by using one group of photons for the sample measurement and the other group for the reference measurement. This is the principle of noise suppression utilized in this study. The achievable degree of noise suppression here is determined by the loss of photons in the measurement, because it destroys the balance of the number of photons in the two groups. Among the various loss sources, the absorption of photons by the sample is unavoidable in absorption spectroscopy, and it sets the ultimate limit to the degree of noise suppression achievable by this method. This ultimate noise suppression over the shot-noise limit (Eq. (3)) is given in terms of the absorbance $A$ of the sample as (see Supplementary Note 6 for the derivation)

4 $$\frac{\delta A}{\delta {A}_{{SN}}}=\sqrt{\frac{{10}^{A}-1}{{10}^{A}+1}}.$$

Second, the emission directions of the paired photons are correlated because of the momentum conservation in the SPDC process.

5 $${{{{{{\bf{k}}}}}}}_{p}={{{{{{\bf{k}}}}}}}_{s}+{{{{{{\bf{k}}}}}}}_{i}.$$

Here, ${{{{{{\bf{k}}}}}}}_{p}$ is the wave vector of the ultraviolet pump light, and ${{{{{{\bf{k}}}}}}}_{s}$ and ${{{{{{\bf{k}}}}}}}_{i}$ are the wave vectors of the generated paired photons, usually denoted as signal and idler. With the Type-I phase-matching condition, this momentum conservation results in ring-shaped emission of the photon pairs (corresponding experimental data will be shown in Fig. 2a), where the photons in the upper half of the ring are paired with those in the lower half of the ring. Thus, it is possible to split the paired photons into two by separating the ring into the upper half and the lower half with the use of, for example, a carefully positioned rectangular mirror that reflects only the upper half of the ring. These two groups of photons obtained in this manner contain exactly the same number of photons, as described in the previous paragraph.

Third, the paired photons have correlated frequencies because of the energy conservation in the SPDC process.

6 $${\omega }_{p}={\omega }_{s}+{\omega }_{i}.$$

Here, ${\omega }_{p}$ is the (angular) frequency of the ultraviolet pump light, and ${\omega }_{s}$ and ${\omega }_{i}$ are those of the generated paired photons. Since ${\omega }_{p}$ is determined by the ultraviolet laser used in the experiment, a down-converted photon with a certain frequency $\omega$ has a partner photon whose frequency is uniquely determined to be ${\omega }_{p}-\omega$. Thus, when the sample and reference spectra are recorded with frequency-resolved detection, the absorbance at a frequency $\omega$ can be obtained by normalizing the number of photons at $\omega$ in the sample measurement by the number of photons at ${\omega }_{p}-\omega$ in the reference measurement. By performing this analysis for various values of $\omega$, the absorbance can be evaluated simultaneously at multiple wavelengths at the sub-shot-noise level, i.e., broadband multichannel sub-shot-noise absorption spectroscopy can be realized. We note that the frequency of the photons in the sample measurement ($\omega$) and that in the reference measurement used for the normalization (${\omega }_{p}-\omega$) are in general different, except at the degenerate frequency ($\omega ={\omega }_{p}-\omega ={\omega }_{p}/2$). This is in sharp contrast to conventional absorption spectroscopy, where a sample measurement performed at a certain frequency is normalized by a reference measurement performed at the identical frequency.

### Experimental setup

**Fig. 1:**
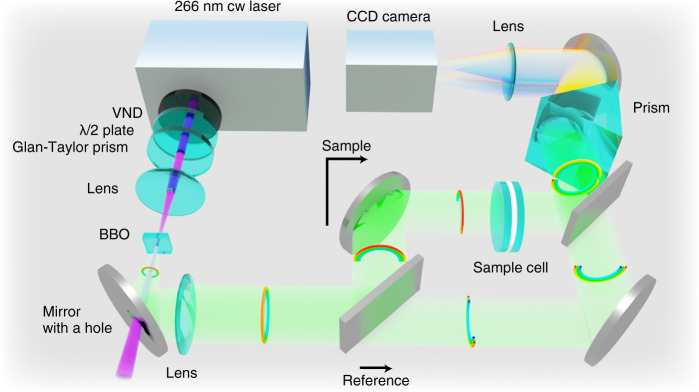
Experimental setup for broadband sub-shot-noise absorption spectroscopy with multichannel detection. The rainbow-colored rings schematically show the propagation of the entangled photon pairs generated by the SPDC process in BBO. Note that the ring is split into the upper and lower halves by a rectangular mirror positioned at an appropriate height. (BBO β-barium borate, CCD charge coupled device, VND variable neutral density filter).

As an experimental realization of broadband multichannel sub-shot-noise absorption spectroscopy described in the previous section, we constructed a setup shown in Fig. 1. The light source is a deep ultraviolet continuous wave (cw) laser providing 266 nm light, and the beam is focused into a BBO crystal to generate entangled photon pairs with the Type-I SPDC process. The resultant ring-shaped emission is separated into two by reflecting only the upper half of the ring using a rectangular mirror. Then, the upper half is sent to a sample cell containing a sample solution, and the lower half is used as a reference. These two portions of the down-converted photons are spectrally dispersed by a prism and are imaged onto a charge-coupled device (CCD) camera, eventually yielding a sample spectrum and a reference spectrum.

In this setup, we took care to minimize the loss of photon pairs, because the loss of photons in the system substantially degrades the noise suppression based on the entanglement of photons, as mentioned in the previous section and discussed thoroughly in Supplementary Note 5. The detection efficiency of the photon pairs in this setup is estimated to be approximately 70%. (Due to the wavelength dependence of the quantum efficiency of the CCD camera, we expect the detection efficiency to vary by ~5% between 450 nm and 650 nm, with the maximum efficiency located at ~530 nm.)

### Characterization of the entangled photon pairs

**Fig. 2:**
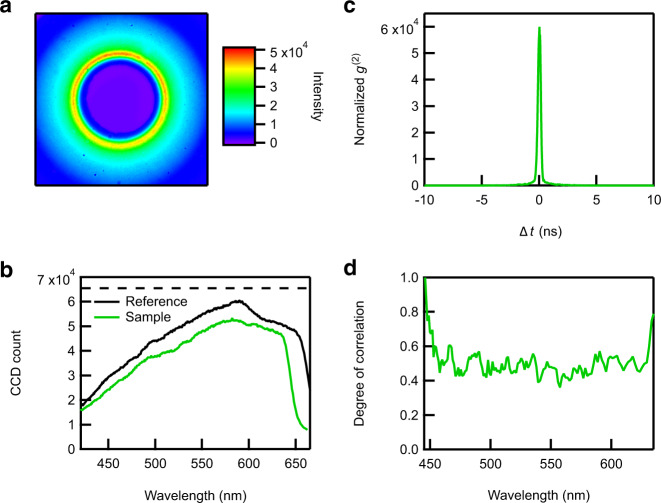
Characterization of the entangled photon pairs. **a** Emission pattern of the photon pairs generated by the SPDC process. **b** Spectrum of the photons sent to the reference path (black curve) and those to the sample path (green curve). The sample used in this measurement was neat DMSO (no absorption within this spectral range). The horizontal broken line indicates the saturation level of the CCD camera. **c** *g*^(2)^ curve showing the temporal correlation of the photons in the reference and the sample paths. The curve is normalized so that the value becomes unity at sufficiently large ${\Delta} t$ where no photon correlations are expected. **d** Degree of correlation defined by Eq. (7), which shows the correlation of photons in the sample and reference paths at each wavelength.

Figure 2a shows the emission pattern of the photon pairs generated by the Type-I SPDC process (see Supplementary Note 1 for the details of the measurement). As mentioned already, it has a ring-shaped emission pattern due to the momentum conservation in the SPDC process (Eq. (5)), where the paired photons appear on the opposite sides of the ring.

After splitting the emission into the lower and upper halves of the ring, we measured the spectra of each part of the emission as shown in Fig. 2b. The green curve corresponds to the upper half of the ring (used for the sample measurement), and the black curve to the lower half (used for the reference measurement). The spectra show that the photons have a broad bandwidth spanning from 400 nm to 650 nm. Because the red side of the spectrum is clipped by the finite size of the optics in the detection path, the true bandwidth is even broader. The exposure time for obtaining these spectra was 375 msec, which was limited by the saturation level of the CCD shown with the horizontal broken line in the figure. The total photon detection rate in the spectral range between 425 nm and 625 nm was $3.4\times {10}^{7}$ photons/sec for the sample measurement and $3.9\times {10}^{7}$ photons/sec for the reference measurement. All the absorption measurements reported in this paper were performed under this experimental condition.

Before performing absorption measurements, we first measured the temporal correlation between the photons in the sample and reference paths to confirm that they are indeed paired. We sent them to two avalanche photodiodes (APDs) connected to a time-correlated single photon counting (TCSPC) board (see Supplementary Note 1 for the details of the measurement) and measured their intensity correlation. In this measurement, the photon detection rate on each APD was attenuated to approximately $2\times {10}^{3}$ photons/sec by using a very low ultraviolet laser power of 1 μW to generate entangled photon pairs (see Supplementary Note 7 for the same measurement performed at different ultraviolet laser powers). The obtained *g*^(2)^ (second-order coherence; ${g}^{\left(2\right)}\left(\Delta t\right)=\left\langle I\left(t\right)I\left(t+\Delta t\right)\right\rangle /\langle I\left(t\right)\rangle \langle I\left(t+\Delta t\right)\rangle$, where $I\left(t\right)$ is the intensity of light at time $t$) exhibits a large coincident count at $\Delta t=0$ as shown in Fig. 2c, which indicates that two photons are very frequently detected simultaneously by the two APDs. This result experimentally proves that the paired photons have been successfully split into the two paths.

We have further confirmed the correlation between the photons in the two paths by evaluating the degree of correlation (also known as the noise reduction factor) defined as^10,14,15^

7 $$\left({{{{{\rm{Degree}}}}}}\; {{{{{\rm{of}}}}}}\; {{{{{\rm{correlation}}}}}}\right)=\frac{{{{{{\rm{Var}}}}}}\left({N}^{S}\left(\omega \right)-{N}^{R}\left({{\omega }_{p}}-\omega \right)\right)}{\left\langle {N}^{S}\left(\omega \right)+{N}^{R}\left({{\omega }_{p}}-\omega \right)\right\rangle }.$$

Here, ${N}^{S}\left(\omega \right)$ and ${N}^{R}\left(\omega \right)$ are the number of photons with the frequency $\omega$ in sample and reference paths, respectively, and ${{{{{\rm{Var}}}}}}({N}^{S}(\omega )-{N}^{R}({\omega }_{p}-\omega))$ is the variance of ${N}^{S}(\omega)-{N}^{R}({\omega }_{p}-\omega)$. We note that ${N}^{R}$ is evaluated at the frequency ${\omega }_{p}-\omega$ because of the frequency correlation of paired photons shown in Eq. (6). The degree of correlation defined in this manner is equal to or larger than unity when there is no correlation between the photons in the sample and reference paths, whereas it approaches zero as the correlation between the two becomes stronger. We have determined the degree of correlation in our experiment by repeatedly measuring the sample and reference spectra such as the spectra shown in Fig. 2b. The obtained result is shown in Fig. 2d. The degree of correlation below 1 is a clear indication that the photons in the sample path at the frequency $\omega$ are indeed correlated with those in the reference path at the frequency ${\omega }_{p}-\omega$.

### Sub-shot-noise absorption measurements

**Fig. 3:**
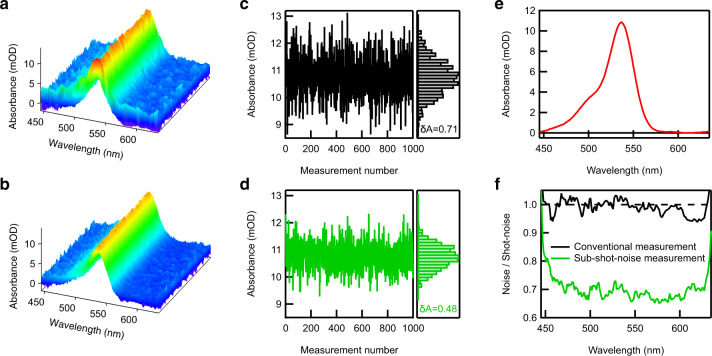
Sub-shot-noise absorption measurements. **a, b** Absorption spectra of 518 nM R6G solutions in DMSO measured by conventional (**a**) and sub-shot-noise (**b**) absorption spectroscopy. The same measurement was repeated 1000 times, and each figure shows 1000 spectra obtained. **c, d** The left part of the figure shows the absorbance value at the absorption maximum (538 nm) evaluated for 1000 spectra measured consecutively. **c** was obtained from the conventional absorption spectra in **a**, and **d** was obtained from the sub-shot-noise absorption spectra in **b**. The right part of the figure shows the corresponding histogram and the noise $\delta A$ [mOD] determined from Eq. (2). **e** Averaged spectrum obtained from the 1000 sub-shot-noise absorption spectra in **b**. **f** Noise $\delta A$ contained in the measured absorption spectra at each wavelength normalized by the shot noise ($\delta {A}_{{SN}}$ in Eq. (3)). The black curve was obtained from **a** and the green curve from **b**. The horizontal broken line corresponds to the shot-noise limit.

With the correlation between the photons in the sample and reference paths established, we utilized them to measure absorption spectra of 518 nM rhodamine 6 G (R6G) solution in dimethyl sulfoxide (DMSO). The measurement was repeated 1000 times to examine the noise contained in the measured spectra. For comparison, we also obtained conventional absorption spectra using the same experimental data set by calculating absorbance from the sample and reference signals detected in different exposures (see Supplementary Notes 2 and 3 for the details of the analysis procedure). In this way, we were able to obtain conventional and sub-shot-noise absorption spectra under exactly the same experimental condition, including the number of photons used in the measurements, which allowed us to have a fair comparison between the two. Figure 3a and b show the obtained conventional and sub-shot-noise absorption spectra, respectively. The two sets of absorption spectra show essentially the same spectral feature, that is, both of them show an absorption maximum at 538 nm (see also the averaged spectrum in Fig. 3e). However, a careful inspection of the spectra reveals that the sub-shot-noise spectra in Fig. 3b contain considerably less noise than the conventional counterpart in Fig. 3a. This can be confirmed in Fig. 3c and d, where the peak absorbance at 538 nm in the 1000 conventional (Fig. 3c) and sub-shot-noise (Fig. 3d) spectra are plotted with their histograms. The noise ${{{{{\rm{\delta }}}}}}A$ evaluated from Eq. (2) is shown in each figure, which quantitatively shows that the noise in the sub-shot-noise absorption spectrum at 538 nm is 32% less than the conventional counterpart. In exactly the same manner, the noise at other wavelengths is evaluated using the experimental data in Fig. 3a and b. The noise obtained in this manner was normalized by the shot noise ${{{{{\rm{\delta }}}}}}{A}_{{SN}}$ evaluated from Fig. 2b using Eq. (3), and the resultant normalized noise $\delta A/{{{{{\rm{\delta }}}}}}{A}_{{SN}}$ was plotted in Fig. 3f. As expected, the noise in the conventional absorption spectra (black curve in Fig. 3f) is approximately at the shot-noise limit. On the other hand, the noise in the sub-shot-noise absorption spectra (green curve in Fig. 3f) is as much as 30% below the shot-noise limit in a broad bandwidth spanning from ~450 to ~650 nm. This accords well with a theoretical prediction that takes account of the total photon detection efficiency of ~70% (see Supplementary Note 5 for the result of the numerical simulations). The obtained result proves that broadband multichannel sub-shot-noise absorption spectroscopy is successfully realized in our experiment. We note that the theoretical bound in Eq. (4) predicts the noise reduction by 89% below the shot-noise limit (when $A=10$ mOD) or even 97% below the shot-noise limit (when $A=1$ mOD) in case of perfect detection efficiency. Therefore, in principle, we have a lot of room for further suppression of the noise by ameliorating the detection efficiency.

### Superresolution concentration measurement

**Fig. 4:**
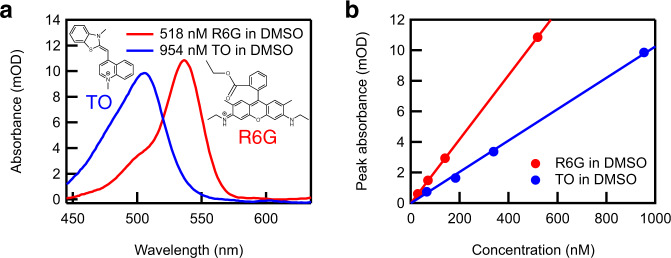
Absorption spectra of R6G (rhodamine 6G) and TO (thiazole orange) and the concentration dependence of the peak absorbance. **a** Absorption spectra of 518 nM R6G solution in DMSO (red curve) and 954 nM TO solution in DMSO (blue curve). The molecular structures of R6G and TO are also shown. **b** The peak absorbance of R6G (red) and TO (blue) solutions in DMSO as a function of their concentrations. The filled circles are the experimental data points obtained by averaging 1000 sub-shot-noise absorption spectra, and the solid lines are the linear fits. The statistical uncertainty of each data point is approximately 0.02 mOD, and the coefficient of determination ${R}^{2}$ for the linear fits^28^ is ${R}^{2}=1.0$ for R6G and ${R}^{2}=0.97$ for TO, indicating a strong linear relationship between the absorbance and the concentration.

To demonstrate how the noise reduction using entangled photon pairs is beneficial for absorption spectroscopy, we applied broadband multichannel sub-shot-noise absorption spectroscopy for identification and quantification of chemical species in highly diluted solutions. As an exemplary case, we chose R6G and thiazole orange (TO) in DMSO. As shown in Fig. 4a, R6G and TO can be clearly distinguished from each other because of their spectral difference. Furthermore, the concentration of each species can be determined from the intensity of the absorption, provided that the relationship between the absorbance and the concentration is known a priori. Figure 4b plots the peak absorbance of R6G and TO in highly diluted solutions with known concentrations (28, 70, 140, and 518 nM for R6G solutions and 65, 183, 338, and 954 nM for TO solutions). The measured absorbance clearly shows a linear relationship to the concentration, as expected.

Using the quantitative spectral information provided in Fig. 4, we determined the concentration of R6G and TO in binary mixture solutions based on absorption spectra, assuming that there are no specific intermolecular interactions among R6G and TO molecules. Although there are several ways to analyze absorption spectra with multiple spectral components, we performed the analysis using deep learning, where the spectral input is processed by an artificial neural network comprising two layers of densely connected neurons. We used the measured absorbance values at 84 wavelengths as the input and obtained the concentrations of R6G and TO as the output. As a preparation, the neural network was trained using the 8 sets of R6G and TO spectra at different concentrations corresponding to the 8 filled circles in Fig. 4b, as well as their linear combinations. (Note that each set contains 1000 spectra, as shown in Fig. 3.) Subsequently, the trained neural network was used to estimate the concentrations of R6G and TO from the absorption spectra of binary mixture solutions. The advantage of this approach is that we do not need to explicitly examine the spectral shape of each species because it is automatically taken care of by the artificial neural network. Thus, exactly the same analysis procedure is applicable to much more complicated spectra such as vibrational spectra, as is already reported in the literature^17^.

**Fig. 5:**
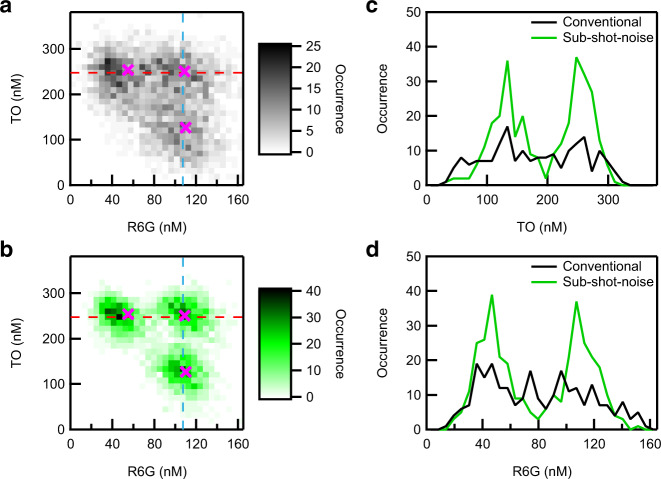
Superresolution concentration measurements. **a, b** 2D histograms of the R6G and TO concentrations in three binary mixture solutions estimated by conventional (**a**) and sub-shot-noise (**b**) absorption spectroscopy. For each binary mixture solution, 1000 absorption spectra were measured, and the concentrations of R6G and TO were estimated from each spectrum. The pink crosses indicate the concentrations of R6G and TO set by the sample preparation process. **c** Vertical cross-sections of the 2D histograms along the blue lines in **a** and **b**. The black curve was obtained from **a** and the green curve from **b**. **d** Horizontal cross-sections along the red lines in **a** and **b**. The black and green curves were obtained from **a** and **b**, respectively.

We prepared three binary mixture solutions with different concentrations of R6G and TO. The concentration combinations of R6G and TO in the three solutions were (55 nM, 254 nM), (110 nM, 127 nM), and (109 nM, 251 nM), where the former number in the parenthesis corresponds to the R6G concentration and the latter to the TO concentration. In these solutions, we made the concentration of TO higher than that of R6G, because TO has a lower absorption cross-section compared with R6G as can be seen from Fig. 4b. For each binary mixture solution, we measured 1000 absorption spectra (3000 spectra in total), and the concentration combination of R6G and TO was evaluated from each of them using deep learning. The 3000 estimated concentration combinations obtained are visualized in two-dimensional (2D) histograms shown in Fig. 5a and b. (Fig. 5a was obtained from conventional absorption spectra, and Fig. 5b from sub-shot-noise absorption spectra). In these 2D histograms, the three crosses indicate the concentration combinations of the binary mixture solutions set by the sample preparation process.

As shown in this figure, the conventional absorption spectroscopy cannot resolve the three binary mixtures (Fig. 5a), because the precise evaluation of the spectral shape and intensity is hindered by the noise present in the spectra. This noise is shot-noise limited as can be seen from the black curve in Fig. 3f, and hence this is the fundamental limit to the concentration and species resolution achievable with conventional absorption spectroscopy under this experimental condition. Meanwhile, the sub-shot-noise absorption spectroscopy clearly resolves the three binary mixture solutions (Fig. 5b). The strength of sub-shot-noise absorption spectroscopy over the conventional one becomes even more evident by evaluating the cross sections of the 2D histograms in Fig. 5a and b. Figure 5c shows the vertical cross sections along the blue broken lines in Fig. 5a and b. With conventional absorption spectroscopy (black curve), the two binary mixture solutions are not resolved, while sub-shot-noise absorption spectroscopy can clearly resolve them (green curve). The same is true for the horizontal cross section along the red broken lines in Fig. 5a and b (Fig. 5d). These results clearly show that sub-shot-noise absorption spectroscopy enables us to determine the concentration of each species in highly diluted solutions with resolution beyond the fundamental limit of conventional absorption spectroscopy. In this sense, it is possible to say that sub-shot-noise absorption spectroscopy can achieve superresolution in concentration measurements. This is analogous to superresolution microscopy, where the spatial resolution is enhanced beyond the diffraction limit using various tricks such as single-molecule localization^18,19^, selective de-excitation of molecules^20^, deconvolution^21^, or photon antibunching of emission from quantum emitters^22^. In the present study, we break the resolution limit in concentration measurements due to the shot-noise limit of conventional absorption spectroscopy by taking advantage of the photon number correlation of entangled photon pairs.

One might argue that the noise in conventional measurements can be made smaller by using a brighter classical light source, because Eq. (3) shows that the shot noise ${{{{{\rm{\delta }}}}}}{A}_{{SN}}$ decreases as we increase the number of photons used in sample (${N}^{S}$) and reference (${N}^{R}$) measurements. We emphasize that this is not possible in our measurements, because we have already accumulated photons up to the saturation level of our CCD as can be seen from Fig. 2b. Thus, the availability of a brighter classical light source does not enable conventional measurements to achieve a lower noise level. This restriction arising from the saturation may be avoided if we allow for a more frequent readout of the CCD and the subsequent averaging of the resultant multiple spectra, but this possibility is not considered in the present study.

## Discussion

In this study, we developed broadband sub-shot-noise absorption spectroscopy with multichannel detection using entangled photon pairs as the light source. The noise in the measured absorption spectra was suppressed by as much as 30% below the shot-noise limit that is the fundamental limit in conventional absorption spectroscopy using classical light. By taking advantage of this suppressed noise, we demonstrated superresolution measurements of the concentrations of chemical species in highly diluted binary mixture solutions.

Sub-shot-noise absorption spectroscopy developed in this study proves particularly useful when the signal-to-noise ratio of the absorption spectra cannot be improved by simply increasing the number of incident photons in a measurement. There are a number of cases when this situation actually happens. The first case is when the number of incident photons is already limited by the saturation of the detector. This applies to the measurement reported in this study, as can be seen in Fig. 2b. The second case is when the measurement needs to be performed within a very short time. In flow cytometry, for instance, a sample quickly passes through the observation volume, and the measurement needs to be performed within a limited time^23^. The third case is when the sample is easily damaged by photoirradiation^24^. Absorption spectroscopy involves an electronic excitation of the sample molecules, and the chance for those molecules to undergo irreversible chemical reactions increases when they are repeatedly excited by intense incident light. The fourth case is when the excited-state lifetime of the molecules is long. Since the absorption occurs only when the molecules are in the ground state, the absorption saturates when the excitation rate becomes comparable to the relaxation rate from the excited state to the ground state^25^. The consideration here shows that there are various cases when the noise suppression is the only means for improving the signal-to-noise ratio. Thus, we envision that broadband multichannel sub-shot-noise absorption spectroscopy developed in this study finds application in various experiments as a unique technique to improve the signal-to-noise ratio beyond the fundamental limit of the conventional absorption measurements.

## Methods

### Sample

Dimethyl sulfoxide (Special Grade) was purchased from Wako Chemicals. Rhodamine 6 G (purity 99%) and Thiazole Orange (purity ~90%) were purchased from Sigma-Aldrich. All the reagents were used as received without further purifications.

### Broadband sub-shot-noise absorption spectrometer

The experimental setup is schematically shown in Fig. 1. A deep ultraviolet cw laser at 266 nm (Coherent, Azure) was used as the light source for generating entangled photon pairs. After going through a half-wave plate and a Glan-Taylor prism, the vertically polarized deep ultraviolet light was focused into a BBO crystal (*θ* = 44.3 deg, *ϕ* = 0 deg, thickness = 0.5 mm) using a lens (*f* = 300 mm), in which horizontally polarized photon pairs were generated by the Type-I SPDC process. The typical laser power at the BBO position was 2 mW. (Higher laser power resulted in the increase of noise in absorption measurements, and eventually caused a damage to the BBO crystal.) Since the photon pairs are emitted in a ring-shaped direction, it is possible to separate the photon pairs from the ultraviolet pump light using a dielectric mirror with a hole at the center (hole diameter = 5 mm), through which the pump light is discarded. The photon pairs reflected by this mirror were then collimated by an antireflection (AR)-coated achromatic lens (*f* = 100 mm). In order to split the paired photons into two, the upper half of the collimated beam was reflected by a rectangular dielectric mirror. The vertical position of this mirror was precisely adjusted using a micrometer-controlled translation stage. The reflected portion of the beam was sent to the sample cell and was used for the sample measurement, whereas the lower half of the beam that passed below the mirror was used for the reference measurement. Subsequently, both the upper and lower halves of the beam were spectrally dispersed in a prism made of flint glass (F2). A prism was chosen as a dispersive optic, instead of a grating, in order to minimize the loss of the photons during the spectral dispersion. The reflection loss at the prism surface was also minimized by choosing the photon pair polarization to be horizontal. Finally, using an AR-coated achromatic lens (*f* = 150 mm), the two spectrally dispersed beams were imaged onto different positions on a thermoelectrically-cooled charge-coupled device (CCD) camera (Princeton Instruments/Acton, PhotonMAX 512B), yielding sample and reference spectra. The readout speed was set at 5 MHz, and the on-chip multiplication gain of the CCD camera was disabled to minimize the noise. Under this condition, the readout noise was 9.90 photons rms for each pixel, and 1 count on the CCD camera corresponded to the detection of 0.78 photons.

The sample solutions were placed in a sample cell and were used for the broadband multichannel sub-shot-noise absorption measurements. The sample cell was also specially designed to minimize the reflection loss of the incident photons. It consisted of two fused silica windows, with AR-coating on the two outer surfaces. The reflection loss at the two inner surfaces was minimized by using a solvent that was index-matched to the fused silica windows, i.e., DMSO. The thickness of the sample solution was adjusted to be 2 mm by placing a spacer between the two fused silica windows.

### Spectral analysis based on deep learning

The deep learning spectral analysis was performed using TensorFlow with Keras frontend^26,27^. Each of our experimental absorption spectra in the spectral range between 460 and 570 nm consisted of 84 data points, and these 84 absorbance values at each wavelength were used as the input for the artificial neural network. The absorption spectra were normalized beforehand using a common normalization factor so that the maximum absorbance in the entire data set becomes one. This input was fed into a sequential model comprising two densely connected layers. The first layer consisted of 64 neurons with the Sigmoid activation function, and the second layer consisted of two neurons without an activation function (linear activation). The output of the two neurons in the second layer corresponded directly to the concentrations of R6G and TO.

We trained this neural network using the absorption spectra of 28, 70, 140, and 518 nM R6G solutions and 65, 183, 338, and 954 nM TO solutions, whose peak intensities are plotted in Fig. 4b with filled circles. Each solution was measured 1000 times with the exposure time of 375 msec, yielding 8000 absorption spectra in total. In order to augment the amount of training data, we took linear combinations of every two data sets. That is to say, if we write the original absorption spectra as ${s}_{1},{s}_{2},\cdots ,{s}_{8}$, we artificially generated spectra expressed by $c\,{s}_{i}+\sqrt{1-{c}^{2}}\,{s}_{j}$ with $i,{j}=1,2,\cdots ,8$ and $c=1/11,2/11,\cdots ,10/11$. In this way, we prepared 288000 spectra in total to train our neural network.

After this training procedure, the concentrations of R6G and TO were estimated from an absorption spectrum by feeding it into the trained neural network.

## Supplementary information

Supplementary Information

## Data Availability

The data that support the findings of this study are available from the corresponding author upon request.

## Code availability

The custom-made computer programs that were used to analyze the data are available from the corresponding author upon request.
