# Supplementary material for: Superresolution concentration measurement realized by sub-shot-noise absorption spectroscopy
Source: Nat Commun. 2022 Feb 17;13:953. doi: 10.1038/s41467-022-28617-w (PMC8854738; doi:10.1038/s41467-022-28617-w)
Supplement: Supplementary file 1 — Supplementary Information [file 41467_2022_28617_MOESM1_ESM.pdf]

Supplementary Information: Superresolution concentration measurement realized by sub-shot-noise absorption spectroscopy

Korenobu Matsuzaki[1,2*] & Tahei Tahara[1,2]

[1] Molecular Spectroscopy Laboratory, RIKEN, 2-1 Hirosawa, Wako 351-0198, Japan

[2] Ultrafast Spectroscopy Research Team, RIKEN Center for Advanced Photonics, RIKEN, 2-1 Hirosawa, Wako 351-0198, Japan

* e-mail: kmatsuzaki@a.riken.jp

Supplementary Figures 1 - 5

Supplementary Note 1: Optical configurations for measuring the emission pattern and intensity correlation $g^{(2)}$

Supplementary Note 2: Analysis procedure for obtaining a sub-shot-noise absorption spectrum

Supplementary Note 3: Analysis procedure for obtaining a conventional absorption spectrum

Supplementary Note 4: Procedure for evaluating the shot noise in the absorption spectra

Supplementary Note 5: Numerical simulation of the noise suppression

Supplementary Note 6: Theoretical bound for the achievable degree of noise suppression

Supplementary Note 7: Power dependence of the $g^{(2)}$ curves

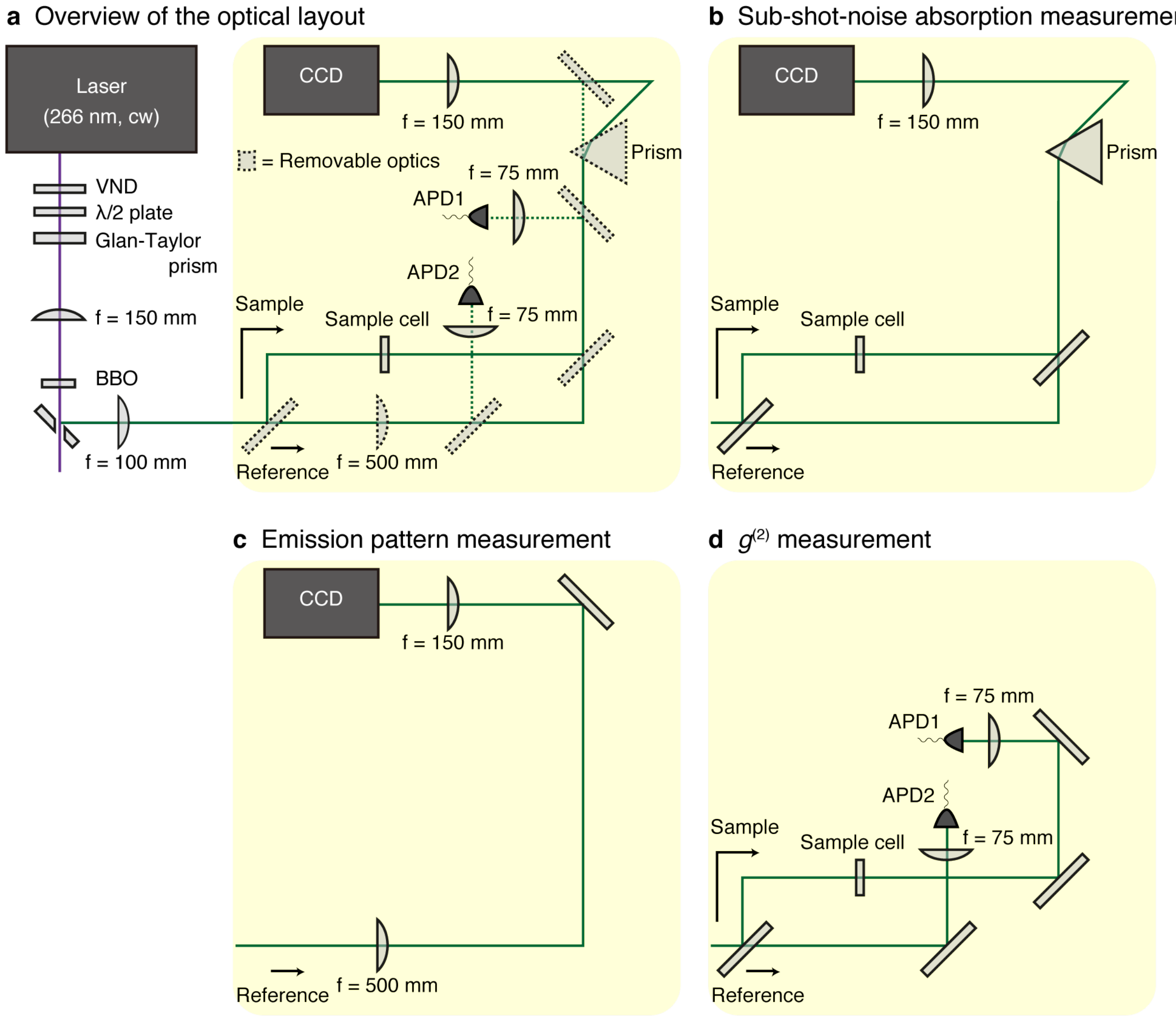

Supplementary Figure 1. Experimental setup. a Overview of the optical layout of the experimental setup constructed in this study. The optics with dotted lines are mounted on a magnetic base or a flip mount, and they can be flexibly removed and reinstalled. Using those removable optics, the yellow part of the setup can be modified with minimum effort of realignment for performing various measurements as shown in the other panels. **b, c, d** Optical configuration for performing the sub-shot-noise absorption measurements (**b**), emission pattern measurements (**c**), and $g^{(2)}$ measurements (**d**).

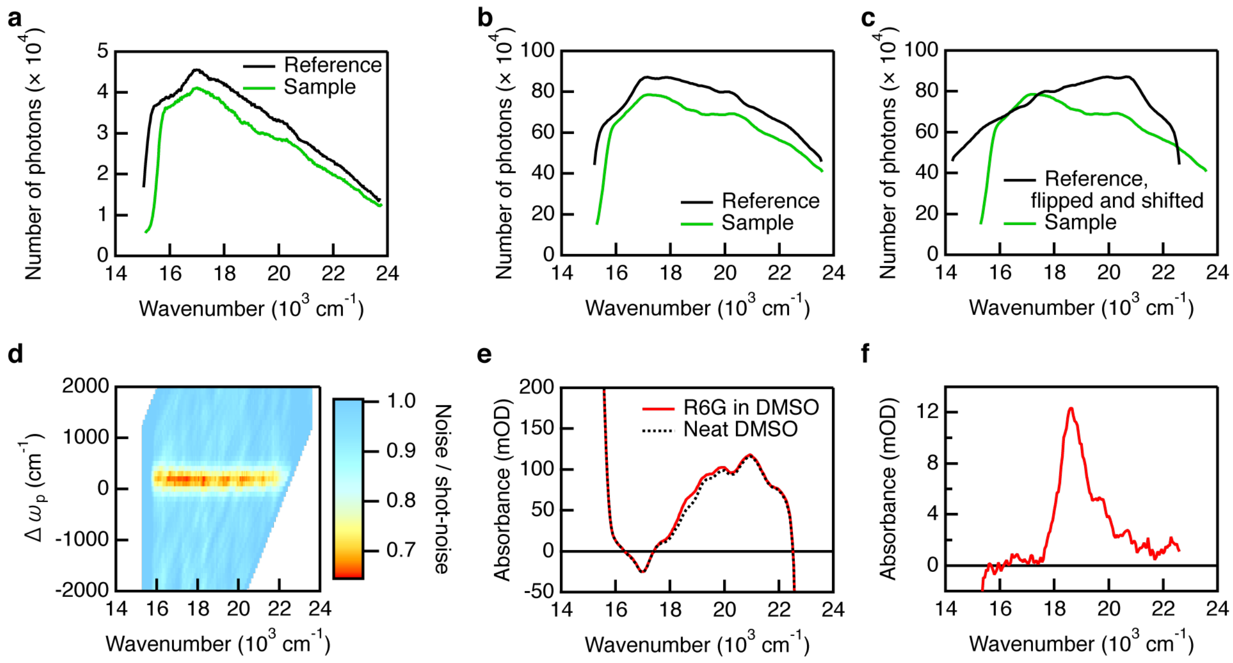

Supplementary Figure 2. Procedure for analyzing experimental data to obtain sub-shot-noise absorption spectra. a Sample (green curve) and reference (black curve) spectra recorded on the CCD camera using 518-nM R6G solution in DMSO as the sample. The spectra are the same as the ones in Fig. 2b in the main text, but the vertical axis is converted to the number of photons and the horizontal axis is also converted to wavenumber. **b** Spectra after the equalization of the horizontal axis. The bin width is $\Delta\omega = 400$ cm^{-1}, and the step size of the center frequency is $\Delta = 50$ cm^{-1}. **c** The black curve shows the reference spectrum flipped and horizontally shifted as described in Supplementary Note 2. The sample spectrum shown by the green curve, which is identical to the one in **b**, can be normalized by simply dividing it with this flipped and shifted reference spectrum. **d** Normalized noise as a function of the pump frequency detuning ($\Delta\omega_p$) introduced in the analysis. The horizontal red line at ~180 cm^{-1} shows a strong noise suppression below the shot-noise limit. **e** Absorption spectra obtained with entangled photon pairs. The red solid curve is obtained from the sample and reference spectra in **c**. The black dotted curve is an absorption spectrum obtained in exactly the same manner using neat solvent (DMSO) as the sample. **f** Baseline-corrected sub-

shot-noise absorption spectrum of 518-nM R6G solution in DMSO. This spectrum is obtained by taking the difference between the red solid curve and the black dotted curve in **e**, and it corresponds to one of the sub-shot-noise absorption spectra shown in Fig. 3b in the main text.

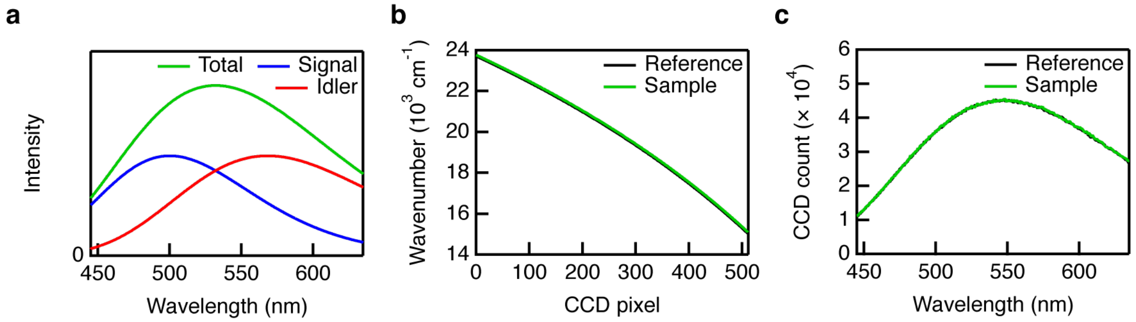

Supplementary Figure 3. Numerical simulation of sample and reference spectra. a Spectra of the photon pairs used in the simulation. The blue curve corresponds to the signal photon, and the red curve to the idler photon. The green curve shows the sum of the two. **b** Experimentally obtained calibration curves that correlate the CCD pixel to the wavenumber. **c** Example of the simulated sample and reference spectra.

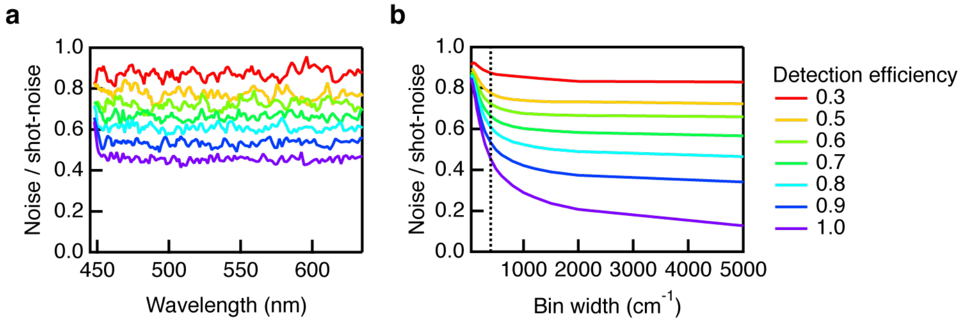

Supplementary Figure 4. Normalized noise obtained from the simulated data. Each color corresponds to a simulation with different detection efficiency as shown to the right. **a** Dependence on the wavelength and the detection efficiency. The bin width is fixed to $\Delta\omega = 400$ cm^{-1}. **b** Dependence on the detection efficiency and the bin width $\Delta\omega$. For each data point, the normalized noise averaged along the wavelength axis is plotted. A vertical dotted line is placed at the bin width of 400 cm^{-1}.

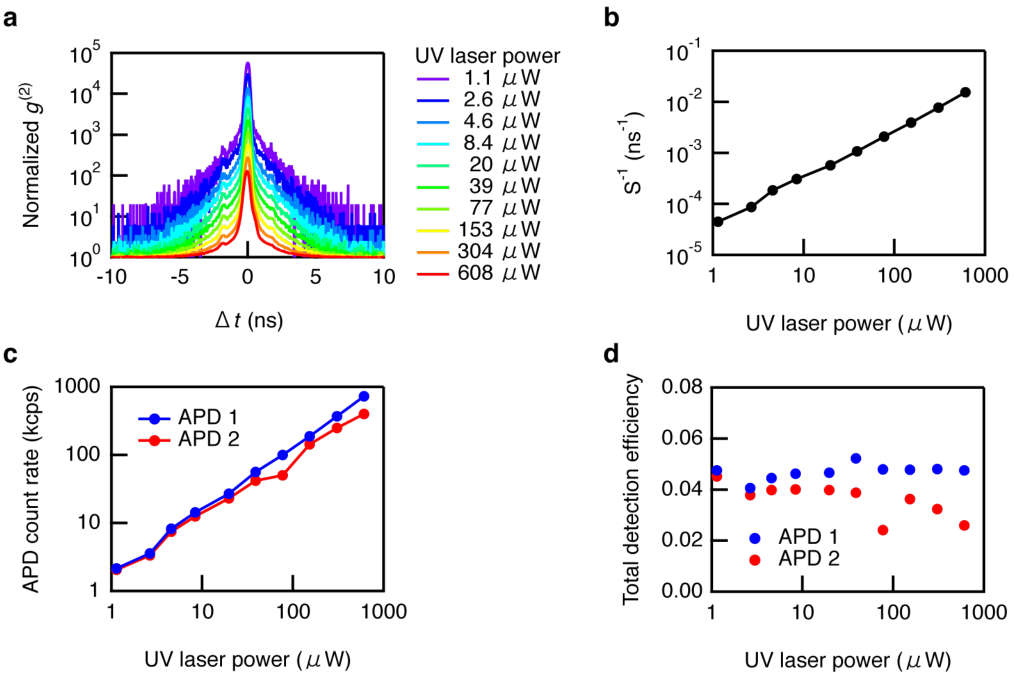

Supplementary Figure 5. Power dependence of the $g^{(2)}$ curves. a $g^{(2)}$ curves of entangled photons pairs measured as a function of the power of the ultraviolet laser that is used for generating entangled photon pairs. Each curve is normalized so that it converges to unity at sufficiently large Δt. **b** Inverse of the area of the bunching peaks ($S = \int \left[g^{(2)}(\Delta t) - 1\right] d\Delta t$) evaluated from each of the $g^{(2)}$ curves in **a**. **c** Count rates on the two APDs measured at each ultraviolet laser power. **d** Total detection efficiency by the two APDs estimated from **b** and **c**. See Supplementary Note 7 for the details.

Supplementary Note 1:

Optical configurations for measuring the emission pattern and intensity correlation $g^{(2)}$

Supplementary Fig. 1a shows the overview of the optical layout of our experimental setup. The optics with the dotted lines are removable, and they enable us to perform sub-shot-noise absorption measurements, emission pattern measurements, and $g^{(2)}$ measurements using the same experimental setup with minimum effort for the realignment, as described below.

Supplementary Fig. 1b shows the optical configuration for performing sub-shot-noise absorption spectroscopy. This is identical to Fig. 1 in the main text.

Supplementary Fig. 1c depicts the optical configuration for measuring the emission pattern of the photon pairs that are generated by the spontaneous parametric down-conversion (SPDC) process, such as the one shown in Fig. 2a of the main text. The f = 500 mm lens is placed 600 mm away from the f = 100 mm lens that collimates the photon pairs generated in the β-barium borate (BBO) crystal. In this way, the back focal plane image of the f = 100 mm lens, i.e., the emission pattern of the photon pairs, can be imaged onto the charge-coupled device (CCD) camera. Typical exposure time for this measurement was 20 seconds.

Supplementary Fig. 1d is the optical configuration for the $g^{(2)}$ measurements. The beams in the sample and reference paths are sent to avalanche photodiodes (APDs) 1 and 2 (idQuantique, id100-MMF50), respectively, via multimode fibers attached to each APD. The temporal correlation between the photon detection events on the two APDs was evaluated using a time-correlated single photon counting (TCSPC) board (Becker and Hickl, SPC-130-EM). To do this, the electronic output of APD1 was sent to the TCSPC board via a router (Becker and Hickl, HRT-82) and was

used as a “start” signal. Meanwhile, the electronic output of APD2 was first sent to a delay generator (Stanford Research Systems, DG535), and was then sent to the TCSPC board to be used as a “stop” signal. The temporal separation between the start and stop signals was evaluated in the TCSPC board, and a $g^{(2)}$ curve was obtained by constructing a histogram of this temporal separation. Typical acquisition time of a $g^{(2)}$ curve was 10 minutes.

Supplementary Note 2:

Analysis procedure for obtaining a sub-shot-noise absorption spectrum

Here we describe how a sub-shot-noise absorption spectrum (such as the one shown in Fig. 3b in the main text) is obtained from the sample and reference spectra recorded on the CCD camera (such as the one in Fig. 2b). As a preparation, the vertical axes of the sample and reference spectra are converted from "count" to "number of photons" using a conversion factor provided by the manufacturer of the CCD camera (0.78 photons/count). Subsequently, the analysis is performed following a three-step procedure comprising (1) the equalization of the horizontal axis spacing, (2) the normalization of the sample spectrum by the reference spectrum, and (3) the baseline correction of the obtained absorption spectrum.

1. Equalization of the horizontal axis spacing

The horizontal spacing of the spectra measured on the CCD camera (Supplementary Fig. 2a) is not even and difficult to be dealt with in the analysis. Therefore, before performing a quantitative analysis of the spectra, it is necessary to make the bin width equal in terms of angular frequency.

More mathematically, if we write the center frequency and the width of the n-th bin as ω_n^{raw} and $\Delta\omega_n^{\mathrm{raw}}$, respectively, the number of photons in this bin (N_n^{raw}) corresponds to the number of photons whose frequency ω is in the range $\omega_n^{\mathrm{raw}} - \Delta\omega_n^{\mathrm{raw}}/2 \le \omega < \omega_n^{\mathrm{raw}} + \Delta\omega_n^{\mathrm{raw}}/2$. For the spectra obtained directly from the experiment, $\Delta\omega_n^{\mathrm{raw}}$ is not constant ($\Delta\omega_n^{\mathrm{raw}}$ varies between 12 and 24 cm^{-1} for the data reported in this study), and ω_n^{raw} is also not linearly dependent on n. The only constraint is $\omega_n^{\mathrm{raw}} + \Delta\omega_n^{\mathrm{raw}}/2 = \omega_{n+1}^{\mathrm{raw}} - \Delta\omega_{n+1}^{\mathrm{raw}}/2$.

With the equalization of the horizontal axis spacing, we redefine the bins so that $\Delta\omega_n$ becomes constant and ω_n becomes linearly dependent on n, i.e., $\Delta\omega_n \equiv \Delta\omega$ and $\omega_n = \omega_{n=0} + \Delta \cdot n$. There

are two factors to consider when we choose a value for the bin width $\Delta\omega$. On one hand, we note that combining multiple bins into one can be done without any ambiguity, whereas splitting a single bin into two is not an obvious process. Therefore, we need to choose a large value for the bin width $\Delta\omega$ in order to minimize the ambiguity arising from the bin splitting (see Supplementary Note 5 and Supplementary Fig. 4 for a more quantitative discussion). On the other hand, the bin width determines the frequency resolution of the resultant absorption spectrum, and it is desirable to keep the bin width small. In order to have a good balance between these two factors, the bin width was set to $\Delta\omega = 400$ cm^{-1} in this study. Meanwhile, the increment of the center frequency of the bins was set to $\Delta = 50$ cm^{-1} to ensure that we have a sufficient number of data points in the obtained absorption spectra. This means that there is a large overlap between the adjacent bins ($\omega_n + \Delta\omega/2 > \omega_{n+1} - \Delta\omega/2$).

In practice, the number of photons in the m-th bin after the bin equilibration (N_m) is evaluated in the following manner. First, we find the bin numbers n_1 and n_2 in the raw spectrum that satisfy $\omega_{n_1}^{\text{raw}} - \Delta\omega_{n_1}^{\text{raw}}/2 \le \omega_m - \Delta\omega/2 < \omega_{n_1}^{\text{raw}} + \Delta\omega_{n_1}^{\text{raw}}/2$ and $\omega_{n_2}^{\text{raw}} - \Delta\omega_{n_2}^{\text{raw}}/2 \le \omega_m + \Delta\omega/2 < \omega_{n_2}^{\text{raw}} + \Delta\omega_{n_2}^{\text{raw}}/2$. Then, the photons in the raw bins with the bin number n satisfying $n_1 < n < n_2$ (number of photons: N_n^{raw}) can be simply combined into N_m, whereas only a portion of the photons in the bins n_1 and n_2 should be added to N_m. Thus, we calculate N_m using the following equation.

$$N_m = \frac{\left(\omega_{n_1}^{\text{raw}} + \Delta\omega_{n_1}^{\text{raw}}/2\right) - (\omega_m - \Delta\omega/2)}{\Delta\omega_{n_1}^{\text{raw}}} N_{n_1}^{\text{raw}} + \sum_{n=n_1+1}^{n_2-1} N_n^{\text{raw}} + \frac{(\omega_m + \Delta\omega/2) - \left(\omega_{n_2}^{\text{raw}} - \Delta\omega_{n_2}^{\text{raw}}/2\right)}{\Delta\omega_{n_2}^{\text{raw}}} N_{n_2}^{\text{raw}}. \tag{S1}$$

This treatment is applied to the sample spectrum as well as the reference spectrum in Supplementary Fig. 2a. The resultant spectra after the horizontal axis equalization are shown in Supplementary Fig. 2b.

2. Normalization of the sample spectrum by the reference spectrum

An absorption spectrum can be obtained by normalizing a sample spectrum by a reference spectrum. This needs to be done by considering the energy conservation condition of the SPDC process (Eq. (6) in the main text). After the equalization of the horizontal axis spacing which is explained in the previous section, the center frequency of the sample spectrum at the n-th bin can be written as $\omega_n^S = \omega_p/2 + \Delta \cdot (n - n_0)$ and the number of photons in this bin as N_n^S. Here, ω_p is the pump frequency (i.e., the frequency of the photon used as the input of the SPDC process), and n_0 is an integer. In a similar manner, for the reference spectrum at the m-th bin, the center frequency and the number of photons are given by $\omega_m^R = \omega_p/2 + \Delta \cdot (m - m_0)$ and N_m^R, respectively. The sample spectrum at the n-th bin needs to be normalized by the m-th bin of the reference spectrum that satisfies $\omega_n^S + \omega_m^R = \omega_p$, which is the energy conservation condition of SPDC. Using the expressions for ω_n^S and ω_m^R given above, we see that this condition is satisfied by choosing $m = m_0 + n_0 - n$. Thus, the absorbance at the frequency ω_n^S can be obtained by comparing N_n^S and $N_{m_0+n_0-n}^R$.

$$A_n = -\log_{10}\left(\frac{N_n^S}{N_{m_0+n_0-n}^R}\right). \tag{S2}$$

The black curve in Supplementary Fig. 2c shows $N_{m_0+n_0-n}^R$ plotted against ω_n^S, and the red curve in Supplementary Fig. 2e shows the absorption spectrum obtained in this manner.

In practice, because of the imperfection of the wavenumber calibration of the sample and reference spectra, we need to finely tune the ω_p value used in the analysis above. To do this, we

decompose ω_p into the true pump frequency $\omega_{p,0} = 37594$ cm^{-1}, corresponding to 266 nm, and the pump frequency detuning $\Delta\omega_p$.

$$\omega_p = \omega_{p,0} + \Delta\omega_p. \tag{S3}$$

Using each $\Delta\omega_p$ value, we obtain absorption spectra, and evaluate the normalized noise at each frequency. The two-dimensional plot in Supplementary Fig. 2d summarizes the noise evaluated in this manner. The figure shows a strong noise suppression at the detuning $\Delta\omega_p \simeq 180$ cm^{-1}, indicating that the proper ω_p value is 37594 + 180 = 37774 cm^{-1}. Therefore, we choose this pump frequency detuning value for obtaining sub-shot-noise absorption spectra.

3. Baseline correction

Finally, a baseline correction is applied to the raw absorption spectrum to obtain the absorption spectrum of the sample. This is done by repeating exactly the same measurement and analysis using a neat solvent as a sample (black dotted curve in Supplementary Fig. 2e), and subtracting this from the raw absorption spectrum obtained in the previous section (red solid curve in Supplementary Fig. 2e). The spectrum obtained after this baseline correction is shown in Supplementary Fig. 2f.

The baseline correction is crucial for sub-shot-noise absorption spectroscopy developed in this study, because the absorbance at ω_n^S is evaluated by normalizing the sample spectrum at ω_n^S by the reference spectrum at $\omega_m^R = \omega_p - \omega_n^S$, where $\omega_n^S \neq \omega_m^R$ except at the degenerate frequency ($\omega_n^S = \omega_m^R = \omega_p/2$). This frequency difference inevitably results in a distortion of the raw absorption spectrum due, for example, to the wavelength dependence of the quantum efficiency of the CCD camera.

Supplementary Note 3:

Analysis procedure for obtaining a conventional absorption spectrum

In Supplementary Note 2, we explained that the absorbance at ω_n^S is evaluated by normalizing the sample spectrum at ω_n^S (number of photons: N_n^S) by the reference spectrum at $\omega_m^R = \omega_p - \omega_n^S$ (number of photons: N_m^R). In the actual measurement, we repeat the same measurement 1000 times, and we therefore have 1000 sets of N_n^S and N_m^R measured at different time $t = l\Delta t$ ($l = 0, 1, \cdots, 999$ and $\Delta t = 375$ msec for the data reported in this paper). We write each of those values as $N_n^S(l^S\Delta t)$ and $N_m^R(l^R\Delta t)$. When $l^S = l^R$, there is a photon number correlation between $N_n^S(l^S\Delta t)$ and $N_m^R(l^R\Delta t)$, and the absorbance can be determined at the sub-shot-noise level by dividing $N_n^S(l^S\Delta t)$ by $N_m^R(l^R\Delta t)$. Meanwhile, when $l^S \neq l^R$, the photon number correlation is absent so that the absorbance obtained from these two values is shot-noise limited. More succinctly, the sub-shot-noise absorbance $A_n^{\mathrm{sub}}(l\Delta t)$ and the conventional absorbance $A_n^{\mathrm{conv}}(l\Delta t)$ are obtained using the following equations in this study.

$$A_n^{\mathrm{sub}}(l\Delta t) = -\log_{10}\frac{N_n^S(l\Delta t)}{N_m^R(l\Delta t)}, \tag{S4}$$

$$A_n^{\mathrm{conv}}(l\Delta t) = -\log_{10}\frac{N_n^S(l\Delta t)}{N_m^R((l+1)\Delta t)}. \tag{S5}$$

In this manner, it is possible to obtain a conventional absorption spectrum under exactly the same experimental condition as the sub-shot-noise absorption spectrum. We note that the noise in the conventional absorption spectrum obtained in this manner is approximately equal to the shot-noise limit as shown by the black curve in Fig. 3f in the main text.

Supplementary Note 4:

Procedure for evaluating the shot noise in the absorption spectra

For a classical state of light, the number of photons at each moment is never constant, and we can only treat it probabilistically. When the average number of photons is N, the probability that there are n photons at a certain moment, denoted as $P_N(n)$, can be modeled by Poisson statistics.

$$P_N(n) = \frac{N^n}{n!} e^{-N}. \tag{S6}$$

The uncertainty of the number of photons can be quantified by evaluating the standard deviation.

$$\delta N = \sqrt{\sum_{n=0}^{\infty} (n - N)^2 P_N(n)} = \sqrt{N}. \tag{S7}$$

This is the shot noise for the number of photons.

The shot-noise limited noise in the absorbance,

$$A = -\log_{10} \frac{N^S}{N^R}, \tag{S8}$$

can be evaluated from the shot noise for the number of photons in the sample measurement (N^S) and in the reference measurement (N^R) based on the error propagation formula. Denoting the noise of N^S and N^R as δN^S and δN^R, respectively, and by assuming that these two noises are not correlated (which is true in conventional absorption measurements), the noise in the absorbance is evaluated to be

$$\delta A = \sqrt{\left(\frac{\partial A}{\partial N^S} \delta N^S\right)^2 + \left(\frac{\partial A}{\partial N^R} \delta N^R\right)^2} = \frac{1}{\ln 10} \sqrt{\left(\frac{\delta N^S}{N^S}\right)^2 + \left(\frac{\delta N^R}{N^R}\right)^2}. \tag{S9}$$

Here, $\ln 10 \equiv \log_e 10$ is the natural logarithm of 10, and we used the relations

$$A = -\log_{10} \frac{N^S}{N^R} = -\frac{1}{\ln 10} \ln \frac{N^S}{N^R}, \tag{S10}$$

and

$$\frac{\partial A}{\partial N^S} = -\frac{1}{\ln 10}\left(\frac{N^S}{N^R}\right)^{-1}\frac{\partial}{\partial N^S}\left(\frac{N^S}{N^R}\right) = -\frac{1}{\ln 10}\frac{1}{N^S}, \tag{S11}$$

as well as

$$\frac{\partial A}{\partial N^R} = -\frac{1}{\ln 10}\left(\frac{N^S}{N^R}\right)^{-1}\frac{\partial}{\partial N^R}\left(\frac{N^S}{N^R}\right) = \frac{1}{\ln 10}\frac{1}{N^R}. \tag{S12}$$

For a shot-noise limited case, we have $\delta N^S = \sqrt{N^S}$ and $\delta N^R = \sqrt{N^R}$. Thus, the noise is given by

$$\delta A_{SN} = \frac{1}{\ln 10}\sqrt{\frac{1}{N^S} + \frac{1}{N^R}}. \tag{S13}$$

This is the shot noise in absorbance, and the normalized noise in Fig. 3f of the main text was obtained by dividing the noise in the experimental absorbance by this δA_{SN}.

Supplementary Note 5:

Numerical simulation of the noise suppression

Numerical simulation was performed to examine how the noise suppression is affected by the two important parameters in sub-shot-noise absorption spectroscopy, i.e., the detection efficiency and the bin width $\Delta\omega$ introduced in Supplementary Note 2. In the simulation, we modeled the probabilistic nature of the SPDC process and the subsequent detection process using random number generation, and simulated the spectra obtained on the CCD camera. Then, we evaluated the extent of the noise suppression by analyzing the simulated spectra in exactly the same manner as for the real experimental data.

1. Generation of simulated spectra

First, we simulate the SPDC process. In this process, a narrow band pump photon at 266 nm is converted into an entangled photon pair (signal and idler photons) with a broad bandwidth. Thus, we assumed that the signal photon has a Gaussian-shaped spectrum centering at 500 nm with FWHM (full width at half maximum) of 5000 cm^{-1} (Supplementary Fig. 3a). Using the probability distribution given by this broadband spectrum, we obtained the frequency of each signal photon detected on the CCD camera with random number generation. Meanwhile, the frequency of the idler photon was determined uniquely from the pump photon frequency and the signal photon frequency following the energy conservation condition given by Eq. (6) in the main text.

The generated photon pair in the SPDC process is then split into two, and one of them is sent to the sample and the other is used as a reference. As explained in the experimental section in the main text, whether the signal photon is used for the sample measurement or for the reference is

determined by its emission direction, which is also a probabilistic process. In our simulation, we modeled this process using another random number generation.

Subsequently, the photons are dispersed in a prism, and each photon hits a frequency dependent position on the CCD camera. The correlation between the photon frequency and the CCD position is taken from the experimentally obtained calibration curves (Supplementary Fig. 3b). Using this calibration curve, a CCD pixel number is uniquely determined for each photon frequency. However, in practice, there is still uncertainty as to which CCD pixel actually detects the photon, because of the finite frequency resolution of the experimental setup. In the actual experiment, this uncertainty is approximately 10 pixels (160 μm in size), meaning that a photon hits an arbitrary pixel within these 10 pixels in each incidence. Thus, in the simulation, we used random number generation to choose one pixel out of the 10 pixels, and assumed that the photon hits the pixel chosen in this manner.

Finally, whether or not the photon is actually detected by that pixel on the CCD camera is also a probabilistic process. It depends on the quantum efficiency of the CCD camera, as well as various other photon losses in the experimental setup. This can be also simulated with a random number generation, treating the total detection efficiency as a parameter. In order to evaluate how the detection efficiency affects the extent of the noise suppression, we performed numerical simulations with the detection efficiency varying from 0.3 to 1.0 (0.3, 0.5, 0.6, 0.7, 0.8, 0.9, and 1.0).

Above, we described how the generation and detection of a photon pair are treated in our simulation. In order to obtain a set of spectra with an intensity comparable to the experimental data, this procedure was repeated many times, until the total number of detected photons reached approximately 3×10^7. The number of photons was converted to the CCD count by dividing it by 0.78, which is the conversion factor provided by the manufacturer of our CCD camera (0.78

photons/count). Finally, the readout noise of the CCD camera (9.90 photons rms for each pixel) was added to the simulated spectrum by again utilizing random number generation. Supplementary Fig. 3c shows the resultant sample and reference spectra. In this manner, we generated 500 sets of simulated spectra, and those spectra were saved in the same file format as the real experimental data. The simulated spectra stored in those files were then analyzed in exactly the same way as for the real experimental spectra.

2. Evaluation of the noise suppression

Using the numerically simulated spectra, we evaluated the normalized noise of the absorption spectra in exactly the same way as for Fig. 3f in the main text (Supplementary Fig. 4a). With the detection efficiency of 0.7 and the bin width $\Delta\omega$ = 400 cm^{-1}, the noise suppression obtained from our simulation is in good agreement with our experimental result (green curve in Fig. 3f). Since the normalized noise appears to be independent of the wavelength, we averaged the noise along the wavelength axis and used this single averaged value to represent the noise in each case. For example, when the detection efficiency is 0.7 and the bin width is $\Delta\omega$ = 400 cm^{-1}, the averaged normalized noise is 0.66 (compared to the shot-noise-limited case).

We repeated the same analysis for different combinations of the detection efficiency and the bin width. The result is summarized in Supplementary Fig. 4b. The figure gives us guidance as to how we can minimize the noise in our experiment. The first strategy is to maximize detection efficiency. This is the reason why we took special care to minimize the loss of photons in our experiment. The figure also suggests that there is still room for further suppression of the noise if we can improve the detection efficiency by future technological breakthroughs. The second strategy is to increase the bin width. However, the bin width determines the spectral resolution in our experiment as we mentioned in Supplementary Note 2, and a larger bin width would mean a deterioration in the

spectral resolution. Therefore, it is important to choose an optimum bin width, with which a large noise suppression can be achieved while the spectral resolution is kept reasonably good. With such a consideration, in our analysis of the experimental data, the bin size was set to $\Delta\omega = 400$ cm^{-1} (vertical dotted line in Supplementary Fig. 4b).

Supplementary Note 6:

Theoretical bound for the achievable degree of noise suppression

As we show in Supplementary Note 5 based on numerical simulations, the degree of noise suppression achieved by sub-shot-noise absorption spectroscopy is limited by the loss of photons in the measurements. Here we derive a theoretical bound for the achievable degree of noise suppression by considering ideal situations without the loss of photons.

There are two types of loss sources. The first one is technical losses due to the imperfection of the optics and the detector used in the experiment, e.g., the reflection loss at the lens surface, nonunity reflectivity of mirrors, and nonunity quantum efficiency of the detector. These are technical issues, and we have a chance to mitigate them by technical improvements in the future. The second one is more fundamental: it is the loss due to the absorption by the sample. This is an unavoidable loss, because it is exactly what we wish to measure in absorption spectroscopy. Thus, the sample absorption ultimately limits the degree of noise suppression achievable by sub-shot-noise absorption spectroscopy.

Below, we evaluate the extent of noise suppression in two situations. In the first situation, we assume that there are no technical losses of photons, and we further assume that the sample shows no absorption of photons. In the second situation, we still assume that the technical losses are completely absent, but we take account of the absorption of photons by the sample. We note that a more general situation that also considers the technical losses is examined in Supplementary Note 5 based on numerical simulations. There, additional sources of noise introduced by other experimental details such as the finite size of the CCD pixels are also discussed.

1. No technical losses, and no photon absorption by the sample

As the most ideal case, we consider the situation where no photons are lost in the entire measurement process. The entangled photon pairs used in sub-shot-noise absorption measurements are generated by spontaneous parametric down-conversion (SPDC), which is a probabilistic process. The probability that n photon pairs are generated is given by the Poisson distribution function $P_N(n)$,

$$P_N(n) = \frac{N^n}{n!} e^{-N}, \tag{S14}$$

with N being the average number of generated photon pairs. Since we now do not consider any loss of photons in the measurement, the number of photons detected in the sample and reference measurements (denoted as n_S and n_R, respectively) is $n_S = n_R = n$, and the absorbance is evaluated to be $A = -\log_{10} \frac{n_S}{n_R} = 0$. Even though the number of photon pairs n fluctuates following the Poisson statistics in Eq. (S14), the absorbance A always takes exactly the same value ($A = 0$) because it has no dependence on n. Therefore, the noise in the absorption spectra completely vanishes in this case.

Although this perfect noise suppression is impressive, it is true only when the sample under investigation does not absorb photons at all, which means that no meaningful absorption spectra can be obtained in this case. Therefore, it is unrealistic and of no practical use.

2. No technical losses, but the absorption by the sample is taken into account

We now consider the case where a portion of photons is absorbed by the sample under investigation. We again write the average number of photon pairs as N, and the number of photon pairs used in a particular measurement as n. Since we still consider no technical losses due to the

imperfection of optics and detector, the number of photons detected in the reference measurement is $n_R = n$.

In the sample measurement, on the other hand, some of the photons are lost due to the absorption by the sample. Writing the absorbance of the sample as A, the probability that a photon is transmited through the sample is 10^{-A}, whereas the probability that a photon gets absorbed is given by $1 - 10^{-A}$. When n photons are irradiated onto the sample, the probability that n_S photons are transmitted through the sample and eventually detected is given by the binomial distribution function $B_n(n_S)$.

$$B_n(n_S) = \frac{n!}{n_S!\,(n - n_S)!}(10^{-A})^{n_S}(1 - 10^{-A})^{n-n_S}. \qquad \text{(S15)}$$

To summarize the result so far, the probability for detecting $n_R = n$ photons in the reference measurement and n_S photons in the sample measurements is given by $P_N(n)B_n(n_S)$. The absorbance $A = -\log_{10}\frac{n_S}{n_R}$ is no longer constant, and its noise δA needs to be evaluated using the probability function $P_N(n)B_n(n_S)$. We note that the noise δA cannot be evaluated based on Eq. (S9), because the noises δN^S and δN^R are correlated in this case.

Before discussing the noise of the absorbance A, we first consider the ratio $T = \frac{n_S}{n_R}$ and its noise δT. The noise δT is defined as the standard deviation of T.

$$\delta T = \sqrt{\langle (T - \langle T \rangle)^2 \rangle} = \sqrt{\langle T^2 \rangle - \langle T \rangle^2}, \qquad \text{(S16)}$$

where $\langle \cdot \rangle$ denotes the statistical average. Using the probability function $P_N(n)B_n(n_S)$, the average value $\langle T \rangle$ can be obtained.

$$\langle T\rangle = \sum_{n=0}^{\infty} P_N(n) \sum_{n_S=0}^{n} B_n(n_S)\frac{n_S}{n}$$
$$= \sum_{n=0}^{\infty} P_N(n) 10^{-A} \sum_{n_S=1}^{n} \frac{(n-1)!}{(n_S-1)!\,(n-n_S)!}(10^{-A})^{n_S-1}(1-10^{-A})^{n-n_S}$$
$$= \sum_{n=0}^{\infty} P_N(n) 10^{-A}\{10^{-A} + (1-10^{-A})\}^{n-1} \tag{S17}$$
$$= \sum_{n=0}^{\infty} P_N(n) 10^{-A} = 10^{-A}.$$

In the very last part, we used the fact that $\sum_{n=0}^{\infty} P_N(n) = 1$. In exactly the same manner, we determine the theoretical expression for $\langle T^2\rangle$.

$$\langle T^2\rangle = \sum_{n=0}^{\infty} P_N(n) \sum_{n_S=0}^{n} B_n(n_S)\left(\frac{n_S}{n}\right)^2 = \sum_{n=0}^{\infty} P_N(n) \sum_{n_S=0}^{n} B_n(n_S)\frac{n_S(n_S-1)+n_S}{n^2}$$
$$= \sum_{n=0}^{\infty} P_N(n)\left[\frac{(10^{-A})^2(n-1)}{n}\sum_{n_S=2}^{n}\frac{(n-2)!}{(n_S-2)!\,(n-n_S)!}(10^{-A})^{n_S-2}(1-10^{-A})^{n-n_S}\right.$$
$$\left.+\frac{10^{-A}}{n}\sum_{n_S=1}^{n}\frac{(n-1)!}{(n_S-1)!\,(n-n_S)!}(10^{-A})^{n_S-1}(1-10^{-A})^{n-n_S}\right] \tag{S18}$$
$$= \sum_{n=0}^{\infty} P_N(n)\left[\frac{(10^{-A})^2(n-1)}{n}\{10^{-A}+(1-10^{-A})\}^{n-2}\right.$$
$$\left.+\frac{10^{-A}}{n}\{10^{-A}+(1-10^{-A})\}^{n-1}\right]$$
$$= \sum_{n=0}^{\infty} P_N(n)\left[(10^{-A})^2 + \frac{10^{-A}(1-10^{-A})}{n}\right].$$

In order to evaluate the second term, we make the following approximation using the formula $(1+x)^{-1} \simeq 1-x$ for $x \ll 1$.

$$n^{-1} = \left[N\left(1+\frac{n-N}{N}\right)\right]^{-1} \simeq N^{-1}\left(1-\frac{n-N}{N}\right) = \frac{2}{N}-\frac{n}{N^2}. \tag{S19}$$

Here, we utilized the fact that $\frac{n-N}{N} \ll 1$ when N is sufficiently large, because n follows the Poisson statistics with the average value of N as shown in Eq. (S14). With this approximation, it is straightforward to perform the summation to obtain

$$
\langle T^2 \rangle \simeq \sum_{n=0}^{\infty} P_N(n) \left[(10^{-A})^2 + 10^{-A}(1 - 10^{-A}) \left(\frac{2}{N} - \frac{n}{N^2} \right) \right]
$$
$$
= (10^{-A})^2 + \frac{10^{-A}(1 - 10^{-A})}{N}. \tag{S20}
$$

Using these equations, we arrive at the theoretical expression for the noise δT.

$$
\delta T = \sqrt{\frac{10^{-A}(1 - 10^{-A})}{N}}. \tag{S21}
$$

This is in agreement with the theoretical expressions given in refs. 12 and 16 in the main text.

With the noise δT determined, we can finally evaluate the noise of absorbance using the error propagation formula.

$$
\delta A = \left| \frac{\partial A}{\partial T} \delta T \right| = \frac{1}{\ln 10} \frac{\delta T}{T} = \frac{1}{\ln 10} \sqrt{\frac{10^A - 1}{N}}. \tag{S22}
$$

Here, the derivative was evaluated using Eq. (S10). In order to examine the degree of noise suppression, we compare this noise with the shot noise δA_{SN}. By setting $N^R = N$ and $N^S = 10^{-A}N$ in Eq. (S13), we have $\delta A_{SN} = \frac{1}{\ln 10}\sqrt{\frac{10^A+1}{N}}$. The noise suppression over the shot-noise limit is then theoretically expressed as

$$
\frac{\delta A}{\delta A_{SN}} = \sqrt{\frac{10^A - 1}{10^A + 1}}. \tag{S23}
$$

This formula shows the theoretical bound for the noise suppression achievable by sub-shot-noise absorption spectroscopy. It predicts a strong suppression of noise when the absorbance A is sufficiently small. In particular, when $A = 0$, the noise completely vanishes, reproducing our conclusion in the previous section.

Supplementary Note 7:

Power dependence of the $g^{(2)}$ curves

1. $g^{(2)}$ curves measured with various ultraviolet laser powers

In Fig. 2c of the main text, we show a $g^{(2)}$ curve as a proof of the temporal correlation of photons in the sample and reference paths. Actually, this $g^{(2)}$ curve is strongly dependent on the power of the ultraviolet laser that is used to generate entangled photon pairs via spontaneous parametric down-conversion (SPDC). In Supplementary Fig. 5a, we show the $g^{(2)}$ curves measured with various ultraviolet laser powers. All the $g^{(2)}$ curves are normalized so that they converge to unity at sufficiently large Δt. We can readily see that the intensity of the bunching peak at $\Delta t = 0$ grows significantly as we decrease the ultraviolet laser power. We note that a log scale is used for the vertical axis in this plot.

To make the observation more quantitative, we evaluated the integrated area of the bunching peak $S = \int \left[g^{(2)}(\Delta t) - 1\right] d\Delta t$, and plotted its reciprocal value S^{-1} against the ultraviolet laser power as shown in Supplementary Fig. 5b. It is readily seen that S^{-1} increases monotonically with increasing ultraviolet laser power. We note that the count rates on the two APDs also increase monotonically as a function of the ultraviolet laser power, as we show in Supplementary Fig. 5c.

2. Origin of the observed power dependence

We now introduce a theoretical model to understand the behavior observed in Supplementary Fig. 5b. We assume that N entangled photon pairs are generated on average within a duration T. If we write the total detection efficiency by the two APDs used in the $g^{(2)}$ measurements as η_1 and η_2, the average count rates on the two APDs are given by $I_1 = \eta_1 N/T$ and $I_2 = \eta_2 N/T$.

The evaluation of $g^{(2)}$ requires us to consider not only the average number of entangled photon pairs but also their number at each moment. The probability that n entangled photon pairs are generated within a duration T is expressed using the Poisson distribution function as $P_N(n) = \frac{N^n}{n!}e^{-N}$. The n photons are sent to each of the two APDs and are detected depending on the total detection efficiencies η_1 and η_2. The probability that at least one of those n photons is detected is expressed as $1 - (1 - \eta_1)^n$ for APD 1 and $1 - (1 - \eta_2)^n$ for APD 2.

We now examine the probability that photons are detected by both of the two APDs. First, we consider the case when the two APDs detect photons simultaneously ($\Delta t = 0$). In this case, the photons are generated simultaneously in the same SPDC process, and the probability is given by

$$\begin{aligned} G^{(2)}(\Delta t = 0) &= \sum_{n=0}^{\infty} \left[1 - \left(1 - \eta_1\right)^n\right]\left[1 - \left(1 - \eta_2\right)^n\right] P_N(n) \\ &= 1 - e^{-\eta_1 N} - e^{-\eta_2 N} + e^{-\eta_1 N} e^{-\eta_2 N} e^{\eta_1 \eta_2 N}. \end{aligned} \tag{S24}$$

Here, we utilized the following relation.

$$\sum_{n=0}^{\infty} x^n P_N(n) = \left[\sum_{n=0}^{\infty} \frac{(xN)^n}{n!}\right] e^{-N} = e^{xN} \cdot e^{-N} = e^{(x-1)N}. \tag{S25}$$

In the meantime, if the two APDs detect photons at different moments ($\Delta t \neq 0$), those photons are generated in separate SPDC processes. The probability in this case is

$$\begin{aligned} G^{(2)}(\Delta t \neq 0) &= \sum_{n_1=0}^{\infty} \left[1 - \left(1 - \eta_1\right)^{n_1}\right] P_N(n_1) \sum_{n_2=0}^{\infty} \left[1 - \left(1 - \eta_2\right)^{n_2}\right] P_N(n_2) \\ &= (1 - e^{-\eta_1 N})(1 - e^{-\eta_2 N}). \end{aligned} \tag{S26}$$

The theoretical expression for the normalized $g^{(2)}$ is then given by

$$g^{(2)}(\Delta t = 0) - 1 = \frac{G^{(2)}(\Delta t = 0)}{G^{(2)}(\Delta t \neq 0)} - 1 = \frac{e^{-\eta_1 N} e^{-\eta_2 N}(e^{\eta_1 \eta_2 N} - 1)}{(1 - e^{-\eta_1 N})(1 - e^{-\eta_2 N})} \simeq \frac{1}{N}. \quad \text{(S27)}$$

In the last part, we made an approximation by assuming that $N \ll 1$.

The derived theoretical expression in Eq. (S27) shows that $g^{(2)}(\Delta t = 0)$ takes a large value for small N, i.e., when few entangled photon pairs are generated due to a low ultraviolet laser power. Intuitively, this is because the probability for detecting two photons generated in separate SPDC processes (i.e., $G^{(2)}(\Delta t \neq 0)$) decreases quadratically as a function of N, while the probability decreases only linearly when the two photons arise from the same SPDC process (i.e., $G^{(2)}(\Delta t = 0)$). This explains the reason why a large bunching peak was observed at $\Delta t = 0$ when the ultraviolet laser power was low as shown in Supplementary Fig. 5a.

We finally consider the theoretical expression for the bunching peak area $S = \int \left[g^{(2)}(\Delta t) - 1\right] d\Delta t$, whose reciprocal value is plotted in Supplementary Fig. 5b. To do this, we recall that our derivation above was based on the number of photon pairs generated within a duration T. This is equivalent to saying that we implicitly considered time bins with the bin width given by T. Thus, the $g^{(2)}(\Delta t = 0)$ value we derived in Eq. (S27) is also valid within this time bin. The bunching peak area S can then be obtained by multiplying $g^{(2)}(\Delta t = 0) - 1 \simeq 1/N$ with the bin width T, i.e., $S = T/N$. This means that its reciprocal value $S^{-1} = N/T$ is proportional to N, explaining the reason why all the data points in Supplementary Fig. 5b are aligned on a straight line.

3. Estimation of the total detection efficiency by the APDs

In Supplementary Fig. 5b, we show the experimentally determined values for the inverse of the bunching peak area $S^{-1} = N/T$. Meanwhile, in Supplementary Fig. 5c, we have the experimental data for the APD count rates $I_1 = \eta_1 N/T$ and $I_2 = \eta_2 N/T$. It is then straightforward to determine the total detection efficiencies η_1 and η_2 by using these two experimental observations.

$$\eta_1 = I_1 S, \qquad \eta_2 = I_2 S. \tag{S28}$$

In Supplementary Fig. 5d, we show the total detection efficiencies determined in this manner. The obtained result consistently shows that $\eta_1 \simeq \eta_2 \simeq 4\%$.

We note, however, that the detection efficiency determined here is irrelevant to the detection efficiency in our sub-shot-noise absorption measurements. While we use a CCD as the detector in the sub-shot-noise absorption measurements, the detectors used in these $g^{(2)}$ measurements are APDs, which have much lower quantum efficiency than the CCD. In addition, we send photons to APDs via optical fibers, and the coupling of photons into the fibers introduces further losses of the photons. These are the reasons for the low detection efficiency determined here.
